# 16S rRNA Gene Amplicon Sequencing Data for Pteris vittata Rhizosphere Soils

**DOI:** 10.1128/mra.01284-22

**Published:** 2023-02-22

**Authors:** Aminu Salisu Mu’azu, Hazzeman Haris, Kamarul Zaman Zarkasi, Nyok-Sean Lau, Amir Hamzah Ghazali

**Affiliations:** a School of Biological Sciences, Universiti Sains Malaysia, Minden, Penang, Malaysia; b College of Science and Technology, Hussaini Adamu Federal Polytechnic, Kazaure, Jigawa State, Nigeria; c Center for Chemical Biology, Sains@USM, Universiti Sains Malaysia, Bayan Lepas, Penang, Malaysia; University of Arizona

## Abstract

Data on the 16S rRNA gene amplicon sequences from Pteris vittata rhizosphere soils are reported. The following phyla were recorded in arsenic-rich soils: *Actinobacteria* (59%), *Proteobacteria* (26%), *Chloroflexi* (17%), and *Acidobacteria* (9%). *Actinobacteria* (45%), *Proteobacteria* (22%), *Chloroflexi* (10%), and *Acidobacteria* (11%) were in natural-mineral soils.

## ANNOUNCEMENT

Pteris vittata, which is also known as Chinese brake, can accumulate large amounts of arsenic (As) in its above-ground biomass, up to 27,000 mg/kg (1, 2), without showing signs of damage, and thus it is called a hyperaccumulator plant; however, dysfunction does start to appear when the concentration of As exceeds 10,000 mg/kg. In contrast, nonaccumulator plants can tolerate As concentrations of only 5 to 100 mg/kg before showing damage (1). Due to this ability, P. vittata has been the subject of much research to understand the mechanism of its resistance to As (1–3). The presence of heavy metals, including As, in soil affects the spread and types of microorganisms found in the soil. These heavy metals are toxic to many microbes, leading to alterations in the properties and diversity of bacterial communities present in the soil. Microorganisms in the soil are influenced by soil pH, which is usually seen as a standard marker of the essential characteristics of microbial populations (4–8). Studies using high-throughput sequencing have revealed new unexplored combinations and diversities of bacterial populations across various soil ecosystems without cultivation (9).

The root system is home to various microorganisms, including bacteria and fungi. These microorganisms are essential in breaking down organic matter, making nutrients available to the plant, controlling plant pathogens, and helping in soil bioremediation (10). Their presence can also alter the soil composition and influence nutrient levels. The soil's high arsenic concentration affects the microbial composition and distribution in areas. Soil samples (200 g) were collected using a standard procedure from areas surrounding the roots (rhizosphere) of P. vittata in six separate locations. Three samples were from arsenic-rich sites (former tin-mining sites) in Perak, Malaysia ([RHT] sample 1 [RHT1], RHT2, and RHT3), and three samples were from Penang, Malaysia (Universiti Sains Malaysia [USM] sample 1 [USM1], USM2, and USM3). Soil tightly attached to the roots of P. vittata was removed using sterile forceps (11, 12). The heavy metal levels in the soil samples were measured using inductively coupled plasma-optical emission spectrometry (ICP-OES). The results revealed that the total arsenic levels in the RHT (arsenic-rich) samples (101 mg/kg to 3,160 mg/kg) were higher than those in the USM samples (5 mg/kg to 6 mg/kg).

**FIG 1:**
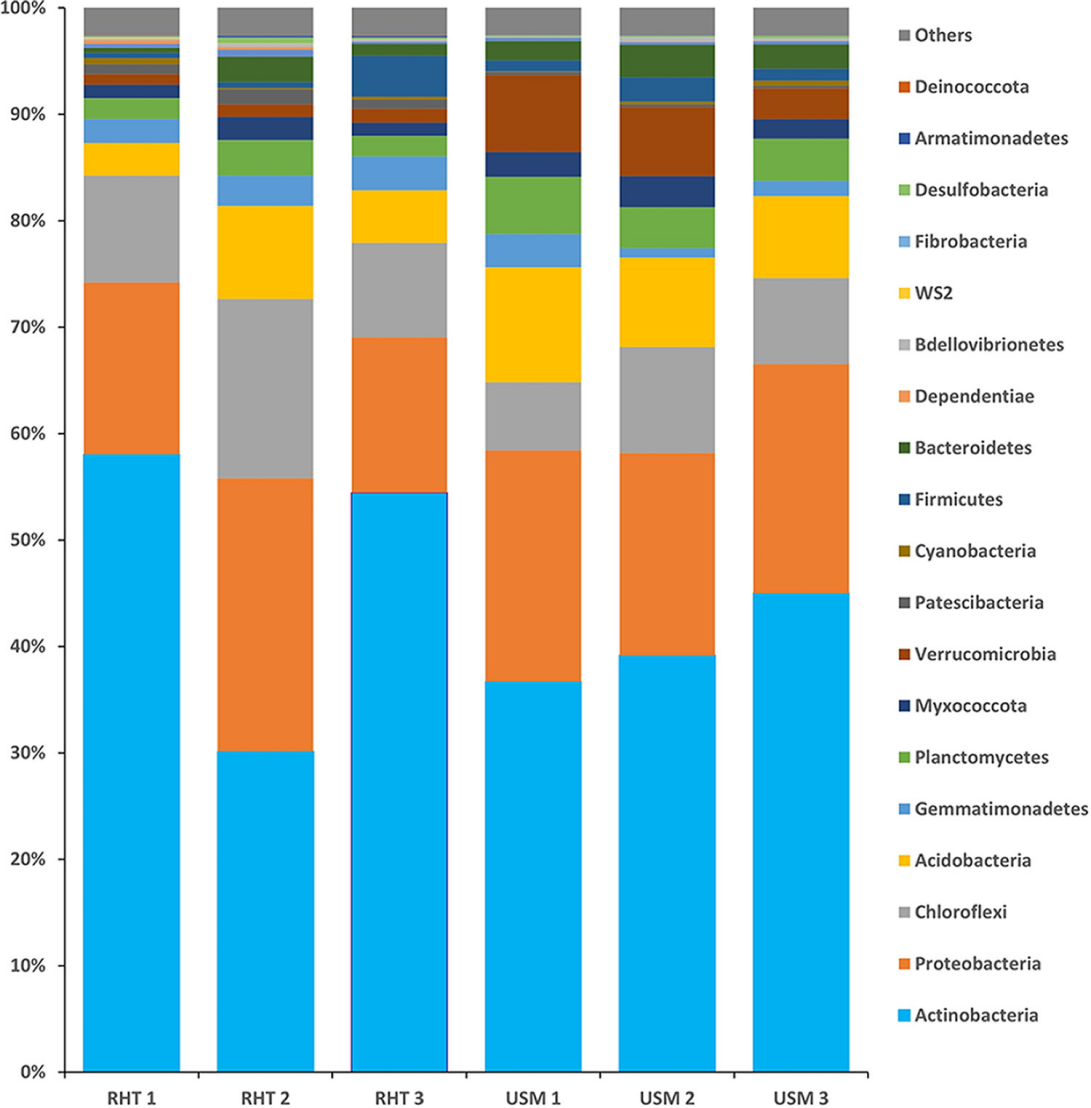
Phylum composition, showing the relative abundance of the top bacterial phyla in arsenic-rich (RHT) and natural-mineral (USM) soils. Each color indicates a different phylum.

The genomic DNA from the soil samples was isolated using the Hi-Yield genomic DNA (soil) extraction kit (Real Biotech Corporation) according to the manufacturer's instructions. The purity of the extracted DNA was examined using a 1% Tris-acetate-EDTA (TAE) agarose gel (13). The 16S rRNA gene amplicon library was prepared following the recommended Illumina protocol, and sequencing was conducted using the MiSeq platform with 300-bp paired-end reads (https://support.illumina.com/downloads/16s_metagenomic_sequencing_library_preparation.html). Locus-specific sequence primers containing overhang adapters were used to amplify a specific segment (V3 to V4) of the bacterial 16S rRNA gene (14). With the help of the BBDuk program from the BBTools package, low-quality reads and sequence adapters were removed from the paired-end reads. The forward and reverse reads were combined using USEARCH v11.0.667 (https://www.drive5.com/usearch) (15). Using UPARSE v11.0.667, the merged reads were grouped into operational taxonomic units (OTUs) based on a *de novo* clustering process with a 97% similarity threshold. The rare OTUs with <2 reads (referred to as doubletons) are often considered spurious and were removed from further analysis. Once the OTUs were created and filtered, PyNAST (16) was utilized to align and generate a phylogenetic tree from a random selection of representative sequences of each OTU. QIIME v1.9.1 was used to match the OTUs to the Silva database for a more accurate taxonomic assignment. The total number of reads recorded for this analysis was 1,086,939 for both the arsenic-rich (RHT) and natural-mineral (USM) soils. The OTUs from the six soil samples were assigned to 38 bacterial phyla, with *Actinobacteria*, *Proteobacteria*, *Chloroflexi*, and *Acidobacteria* as the most abundant bacterial phyla (Fig. 1).

### Data availability.

The amplicon data from this work have been deposited in the Sequence Read Archive (SRA) of the National Center for Biotechnology Information (NCBI) with BioProject accession number PRJNA882671 (Table 1).

**TABLE 1:** Sample descriptions and summary of the 16S rRNA gene amplicon results

Sample type	Sample no.	Sampling location coordinates	SRA accession no.	Sequence length (bp)	Total no. of OTUs	Read length (bp)
As-rich soil	RHT1	5°36.099′N, 101°1.696′E	SRX17998250	243,038	121,487	301
As-rich soil	RHT2	5°36.280′N, 101°2.517′E	SRX17998251	168,137	93,146	301
As-rich soil	RHT3	5°38.460′N, 101°2.767′E	SRX17998252	164,600	90,837	301
Natural soil	USM1	5°21.456′N, 100°18.060′E	SRX17998253	192,975	98,347	301
Natural soil	USM2	5°21.602′N, 100°18.290′E	SRX17998254	161,291	79,958	301
Natural soil	USM3	5°21.341′N, 100°18.048′E	SRX17998255	156,898	70,623	301
